# New Appliances and Things Medical

**Published:** 1898-02-12

**Authors:** 


### NEW APPLIANCES AND THINGS MEDICAL.

[We shall be glad to receive, at our Office, 28 & 29, Southampton Street, Strand, London, W.C., from the manufacturers, specimen of all new preparations and appliances which may be brought out from time to time.]

### WORTH'S PERFECT FOOD.

(Worth's Food Works, Cheltenham.)

This is a malted cereal preparation which, on examination of the sample submitted, is shown to contain all the necessary constituents in a concentrated form for producing a nutritious food for children and invalids. The statement that it is closely allied to the mother's milk cannot be endorsed, considering that the preparation contains at least 68 per cent. of starchy material. In the form of a porridge it would make a nutritious food for infants who have reached the age when amylaceous substances can be digested by them, and for invalids whose digestive organs require an easily assimilable food.

### HUMANIZED MILK (DR. GAERTNER'S PROCESS).

(Friern Manor Dairy Farm, Limited, 20, Farringdon Street, E.C.)

The samples of humanized milk received from the Friern Manor Dairy Farm prepared by Gaertner's process can be safely recommended for use as a substitute for the natural food of the infant when from various causes artificial feeding is indispensable. The milk is contained in sterilised bottles, and a sample, which is stated to have been kept in this manner for three months, was perfectly sweet and wholesome; moreover, no antiseptics could be detected. In addition to preparing humanized milk which shall approximate in chemical composition to mother's milk the Friern Manor Farm Company undertake, without increased charge, the preparation, under medical authority, of milk which shall contain any required proportion of fat, proteids, and milk sugar, and in necessitous cases will diminish the cost by one half upon the certificate of the medical attendant.

### EXTRACT OF BEEF.

(Lipton, City Road, London, E.C.)

The sample of extract of beef submitted to us by Messrs. Lipton is a very concentrated form of beef extract. It contained 63·6 per cent. of meat extractives, including a small proportion of albumen, 20·4 per cent. of mineral matter, and only 16 per cent. of water. It had an agreeable meat flavour, was of a good pale brown colour, and free from all indications of having been over-heated or burnt. In all respects this is an excellent form of extract of beef.

